# Hemostatic efficacy of chitosan-based bandage for closure of percutaneous arterial access sites: An experimental study in heparinized sheep model

**DOI:** 10.2478/v10019-010-0021-0

**Published:** 2010-05-24

**Authors:** Pawanrat Kranokpiraksa, Dusan Pavcnik, Hideaki Kakizawa, Barry T. Uchida, Miran Jeromel, Frederick S. Keller, Josef Rösch

**Affiliations:** Dotter Interventional Institute, Oregon Health Sciences University, Portland, OR, USA

**Keywords:** arterial catheterization, hemostasis, closure devices, hemostatic pads, chitosan-based pad

## Abstract

**Background:**

Most of the presently used percutaneous arterial closure devices (PACD) for hemostasis after interventional vascular procedures are effective, but carry risk of complications by deposition of a foreign body. A new promising externally applied PACD – chitosan-based HemCon Bandage (HCB) was explored in sheep. The HCB hemostatic efficacy and complications occurring with its use were compared to those with the standard manual compression (SMC).

**Material and methods:**

Both superficial femoral arteries (SFA) of 9 heparinized sheep were catheterized with an 8F sheath for 5 minutes. After the sheath withdrawal, hemostasis with the HCB was compared with hemostasis achieved with SMC in the contralateral SFA. Iliac angiograms performed by carotid artery approach determined the hemostasis time.

**Results:**

The HCB use shortened time to hemostasis with a mean time of 6.9 ± 3.9 minutes versus 10.8 ± 2.8 minutes for the SMC (P-value 0.019). Seven SFAs in the HCB group and only 1 SFA in the SMC group exhibited hemostasis in 5 minutes. All nine SFAs using the HCB showed femoral artery patency and demonstrated less hematoma (2/9) than in the SMC group (8/9). No complications developed in the HCB group, one SFA occlusion was seen in the SMC group.

**Conclusions:**

The externally applied HCB in heparinized sheep was safe and effective. It significantly shortened time to hemostasis at the SFA access sites following 8F sheath removal. Proper application of the HCB was necessary to shorten hemostasis and prevent hematoma formation. The HCB should be tested in a clinically controlled study to evaluate its efficacy in humans.

## Introduction

Since the introduction of percutaneous catheterization in 1953^1^, manual compression over the puncture site has been the standard technique for achieving hemostasis in interventional radiology.^2^ With diagnostic angiography using 5F to 6F catheters, manual compression followed by bed rest has been very efficient for achieving hemostasis and has led to less than 1% puncture site complications.^3^ Introduction of therapeutic vascular procedures with the need for 8F and larger introductory sheaths, adjuvant anticoagulation and antiplatelets or thrombolytic therapy has led to an increase in complications. Arterial access site complications as high as 17% have been reported with interventional procedures, some of them requiring corrective surgical treatment.^4^,^5^

Since the early nineties, several types of percutaneous arterial closure devices (PACD) have been introduced to enhance hemostasis after interventional procedures and to decrease the rate of complications. These devices either replace or shorten the time of manual compression at the puncture site.^5^,^6^ Externally applied hemostatic patches and pads that accelerate the hemocoagulation process at the puncture site are one of the newest PACD types. Acceleration of hemostasis is caused by active ingredients of the patches and pads that contain procoagulants that potentiate clot formation.^7^–^9^ All procoagulants require compression for hemostatis, but substantially reduce compression times. Most procoagulants require contact with blood for activation. There have been several clinical studies on the hemostatic efficacy of these patches and pads.^7^–^15^ However, we have found only two experimental reports documenting the hemostatic efficacy of procoagulants in animals. One paper reported exploration of the efficacy of microfibrillar collagen and thrombin applied into the arterial puncture tract in dogs with the help of a balloon catheter.^16^ The other report described a procoagulant (chitosan) installed into the arterial puncture tract in dogs.^17^ Chitosan is a linear polysaccharide derived from chitin commercially extracted from marine arthropod shells. It is composed of positively charged molecules that attract red blood cells and platelets, thereby, promoting hemostasis. We report an exploration of the hemostatic efficacy of chitosan-based HemCon Bandage (HCB) (HemCon Medical Technologies, Portland, OR) and a comparison of these bandages with standard manual compression in a heparinized sheep model. We used sheep for testing since their arteries are similar in size to humans.^18^,^19^ In addition, their coagulation and fibrinolytic systems are closer to those of humans when compared to canine and swine.^20^

## Material and methods

The study protocol was approved by the Institutional Animal Care and Use Committee. Nine female sheep weighing from 56 to 70 kg were used in this study. A cardiac mobile system (GE/ OEC 9800; GE Medical Systems, OEC, Salt Lake City, UT, USA) with digital imaging was used for fluoroscopy and angiography. Digital subtraction angiographies were performed with an injector (Medrad mark Plus, MEDRAD, Inc., Warrendale, PA, USA).

Preparation of animals and their anesthesia were described in previous paper.^18^ After induction of general endotracheal anesthesia, the sheep were placed and secured with their backs on the radiographic table and their hind limbs in moderate abduction. The neck and both groins were shaved and prepped. The right common carotid artery (CCA) was exposed and a 9F, 50 cm long introducer sheath (Cook Medical, Bloomington, IN, USA) was retrogradely inserted into abdominal aorta. A standard dose of heparin (100 IU/kg) was then administered intra-arterially. Activated clotting times (ACT) were recorded at baseline prior to heparin administration, prior to arterial sheath removal and at the end of the procedures. A 5F multiside-hole catheter (Cook Medical) was then introduced through the 9F sheath for selective angiography of the external iliac arteries using an injection of 16 ml of Omnipaque (IOHEXOL 300 mg 1/mil, GE Healthcare, Princeton, NJ, USA) in 2 seconds.

The access sites in each animal were the superficial femoral arteries (SFA). One SFA received treatment with the HCB applied with manual compression and the contralateral SFA served as a control with the use of SMC. The sequences of the puncture sites and treatment modes were randomized. With selective iliac angiography, a road map image was created and SFA diameter was measured. Single wall access of the SFA was done under road map guidance with the 21 gauge needle of the micropuncture set (Cook Medical). An 8F sheath was then placed into the artery and left there for 5 minutes. During the sheath removal and prior to the use of the HCB 2″ X 2″ in size, mild nonocclusive pressure was first applied above the skin puncture site. After the sheath was completely removed, a small amount of blood was first allowed to seep on the skin access site to contact the bandage and initiate hemostasis. The bandage was then applied with digital nonocclusive pressure. In the control SFA, significant digital pressure was applied during the sheath removal to prevent blood penetration through the puncture tract, as used in clinical practice. Manual pressure was held in both the treatment and control groups for 5 minutes. Angiography was done immediately after pressure relief to confirm hemostasis. If angiography showed extravasation, compression was continued for a further 2.5 minutes. Angiography was then repeated every 2-1/2 minutes until no evidence of bleeding was seen. Compression was reapplied in the interval between repeat angiographies. Lack of extravasation was the endpoint. The study then proceeded on the contralateral SFA. Finally, angiography of each side was performed at about 30 minutes after cessation of bleeding to check the patency and status of the SFA. The access sites were then checked for hematomas defined as loss of definition of the fossa subinguinalis and raised appearance of the skin. The degree of hematoma at the groin was graded: 0 = no hematoma, 1 = slight and 2 = significant. Groin area fullness with prominence <1 cm was considered slight hematoma. At the end of the study, the animals were euthanized.

### Statistical analysis

Data were recorded into a worksheet (Excel 2007, Microsoft, Redmonds, WA, USA) and summary statistics (mean and standard deviation) were calculated. Student’s t-test was used to determine if there was a statistically significant difference between the control and treatment with regard to achieving hemostasis. A value of P<0.05 was considered significant.

## Results

**FIGURE 1:**
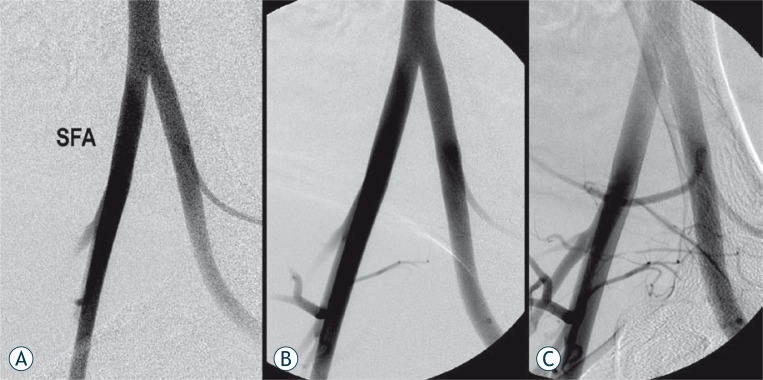
Hemostatic control with the chitosan-based bandage. A - Baseline angiogram prior to the superficial femoral artery (SFA) puncture. B - Angiogram obtained after 8F sheath withdrawal and 5 minutes chitosan-based bandage compression shows complete hemostasis. C - Angiogram obtained 30 minutes after sheath withdrawal shows patent SFA.

SFA diameters ranged from 5.4 to 6.1 mm with all pairs being matched in size. The mean ACT prior to the sheath removal in the HCB group was 404.4 ± 262 seconds and in the control group 371.87 ± 73.1 (p=0.737). The mean time to achieve hemostasis in the HCB group was 6.9 ± 3.9 minutes, while the mean time of the control group was 10.8 ± 2.8 minutes (p=0.019). The results are summarized in Table 1 and 2. In the HCB group, hemostasis at 5 minutes post sheath removal was achieved in 7 of 9 SFAs (77.8%) (Figure 1). The other 2 SFAs exhibited hemostasis at 12.5 and 15 minutes, respectively. One of those two delayed times to hemostasis was equivalent to the control side and the other needed longer time to achieve hemostasis than the control SFA. Hemostasis was obtained at 5 minutes after sheath removal in one of 9 (11.1%) of the control group (Figure 1). Angiography at about 30 minutes after intervention showed no extravasation in either HCB or control group. All SFAs in the HCB group were patent without demonstrable arterial spasm. Eight of 9 (88.9%) SFAs in the control group were patent and spasm was found in 3 (33%) arteries. Two of these SFAs exhibited small defects, presumably thrombi at the access sites. One SFA was occluded. In the HCB group, there were two grade 1 hematomas, while in the SMC group there were eight, two of which were grade 2.

## Discussion

Numerous PACDs are now available for achieving rapid hemostasis at percutaneous arterial access sites after diagnostic or interventional procedures. Madigan *et al*. in 2007 reviewed 14 PACDs.^6^ Other new PACDs are being developed and/or tested.^7^ Based on their principle mechanism of hemostasis, PACDs are categorized into four groups. The first three groups include biodegradable sealing plugs, suture-mediated devices and staple-mediated devices. They are very effective and have been the most frequently used PACDs. However, they have not been used without complications. Their use has been associated with infections due to deposition of a foreign body, bleeding, pseudo aneurysm, arteriovenous fistula and arterial occlusion.^5^,^6^,^18^,^21^ The fourth group of PACDs – patches and pads have recently received close attention. They are topically applied and their procoagulant ingredients accelerate hemocoagulation at the access site without leaving any foreign material behind. Because of their action, they are called “noninvasive” PACDs.^7^ The procoagulant components of the noninvasive PACDs include, among others, bovine thrombin (D-Stat-Dry-Vascular Solutions), poly-N-acetyl glucosamine derived from marine diatoms (Syvek Patch-Marine Polymer Technologies), polyprolate acetate (Clo-Sur Pad – Scion Cardiovascular) and chitosan obtained from exoskeleton of crustaceans (Chito-Seal, Abbot Vascular, HemCon^®^Bandage). Clinical studies of D-Stat-Dry, Syvek Patch, Clor-Sur Pad and Chito-Seal showed that these PACDs applied with compression reduce time to hemostasis after femoral artery catheterization compared with SMC and do not increase the complication rate when using 4–6 F sheaths.^8^,^10^,^12^–^14^

Literature is available on the hemostatic efficacy of the HCB in traumatic animal models, and on HCB use in trauma patients in the military, in emergency departments and during surgery.^22^ However, its efficacy for hemostasis after femoral artery catheterization has not been documented by either experimental or clinical studies. Our experimental study demonstrates that the HCB can be effective in this setting. In our experimental heparinized sheep model using an 8F sheath, the HCB shortened the time to hemostasis to almost half of SMC alone. Hemostasis was achieved with HCB in 7 of 9 puncture sites within 5 minutes, in comparison to only one site in the control group that was within 5 minutes. The two instances where hemostasis with the HCB took the same amount of time or slightly longer time than in the control SFA was due to application of the bandage in a manner similar to that of standard compression where the needed blood to activate the HCB was not allowed to seep through the access tract. This finding strongly reinforces the need for the presence of blood at the access site to initiate the hemocoagulative action of chitosan. The HCB group also demonstrated less hematoma formation than the control group. The higher incidence of hematomas, however, was undoubtedly also related to release of pressure at the access site for performance of angiography before hemostasis could be established. No bleeding was found in both groups 30 minutes after compressions, but in two SFAs small defects suspicious of thrombi were found at the access sites after SMC.

The study limitations include the small sample size and the impossibility of performing a blind study due to the distinctive appearance of the HCB. Another limitation is the absence of data on time to ambulation and long-term efficacy of the closure. Important information about time to ambulation, thus, could not be evaluated. Another study should address these limitations and should also include histopathologic evaluation of the SFAs access site.

## Conclusions

The chitosan-based HCB was effective and shortened the time to hemostasis at SFA access sites following removal of an 8F sheath in heparinized sheep. Proper application of the bandage, however, was necessary to shorten hemostasis and decrease hematoma formation. A controlled clinical study needs to be done for evaluation of hemostatic efficacy of HCB following endovascular interventions in humans.

## Figures and Tables

**FIGURE 2:**
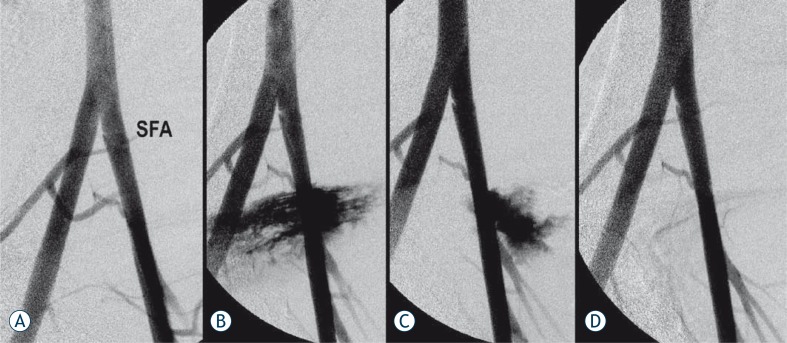
Hemostatic control with the standard manual compression. A - Baseline arteriogram prior SFA puncture. B - Angiogram obtained after 8F sheath withdrawal and 5 minutes standard manual compression shows extensive extravasation from the puncture site. C - After additional 2.5 minutes manual compression (total 7.5 minutes), there is decreased extravasation. D - After additional 2.5 minutes of manual compression (10 minutes total), complete hemostasis is achieved.

**TABLE 1:** Comparison of angiographic findings, ACT values, hemostasis times and post procedure hematomas in 18 punctured superficial femoral arteries, 9 in the HCB and 9 in the control group

	**HCB group**	**Control group**	**p value**

	**n = 9**	**n = 9**	
**SFA diameter (mm)**	5.4 – 6.1	5.4 – 6.1	
**Baseline ACT**	106 – 161	(136 +/− 19.8)	
**ACT prior sheath removal (sec)**	205–1061	267– 480	0.737
	(404.4 +/− 262)	(371.8+/−73.1)	
**Hemostasis time (min)**	5–15 (6.9 +/− 3.9)	5–12.5 (10.8 +/− 2.8)	0.019
**Artery patency at 30 min**	9	8	
**Subcutaneous Hematoma**	2	8 (2 significant)	

**TABLE 2:** ACT values prior to sheath removal, hemostasis times and hematoma presence at puncture site in individual animals

**HCB group**				**Control group**			
**AnimalNo.**	**ACT**	**Hemostasis**	**Hematoma**	**ACT**	**Hemostasis**	**Hematoma**	
1	348	5	0	344	12.5	+	
2	268	5	0	412	7.5	+	
3	447	5	0	257	12.5	+	
4	800	12.5	+	1061	12.5	++	
5	361	15	+	244	12.5	++	
6	480	5	0	205	10	+	
7	262	5	0	260	12.5	+	
8	272	5	0	469	5	0	
9	433	5	0	388	12.5	+	

**Mean+/−**	**372±73.1**	**6.94±3.9**		**404±262**	**10.8±2.8**		

ACT values in seconds, hemostasis time in minutes; Hematoma grades 0 = none, + = minor, ++ = significant
